# Small Bowel Obstruction Caused by Internal Hernia through a Peritoneal Defect of the Pouch of Douglas: Report of a Case and Review of the Literature

**DOI:** 10.1155/2017/9819270

**Published:** 2017-07-05

**Authors:** Pyong Wha Choi

**Affiliations:** Department of Surgery, Ilsan Paik Hospital, Inje University College of Medicine, Goyang, Republic of Korea

## Abstract

Herniation of small bowel through the peritoneal defect of the Pouch of Douglas is extremely rare type of internal hernia, and this type of internal hernia has been described as an entity of perineal hernia. Here, we describe a case of a 26-year-old female without history of abdominal surgery presenting with incarcerated small bowel hernia through a peritoneal defect of the Pouch of Douglas. She visited an emergency department presenting with abdominal pain and distension. Without improvement symptom by conservative management, an operation was performed. During the operation, the distal ileum had been herniated through a peritoneal defect of the Pouch of Douglas, and there were no specific findings on gynecological examination. Reduction of the herniated bowel and primary repair of the peritoneal defect were performed. The case represents a very rare type of internal hernia and provides published cases of hernia through a peritoneal defect of the Pouch of Douglas.

## 1. Introduction

Intestinal obstruction caused by internal hernia is rare, with an incidence of between 0.2% and 0.9% [1]. Diverse types of internal hernias have been reported including paraduodenal, perivesical, intersigmoidal, and transomental hernia [2]. Among internal hernias, pelvic hernias are extremely rare type of hernia, and they are classified into obturator, sciatic, and perineal hernias. Douglas pouch is an extension of the peritoneal cavity between the rectum and the back wall of the uterus, which is also known as the rectouterine pouch [3]. A defect of the Pouch of Douglas may lead to small bowel hernia resulting in incarcerated intestinal obstruction which has been classified as perineal hernia entity [4]. Here, we present a case of a 26-year-old female without history of surgery in which small bowel was herniated through a peritoneal defect of the Pouch of Douglas resulting in incarcerated small bowel obstruction.

## 2. Case Presentation 

**Figure 1:**
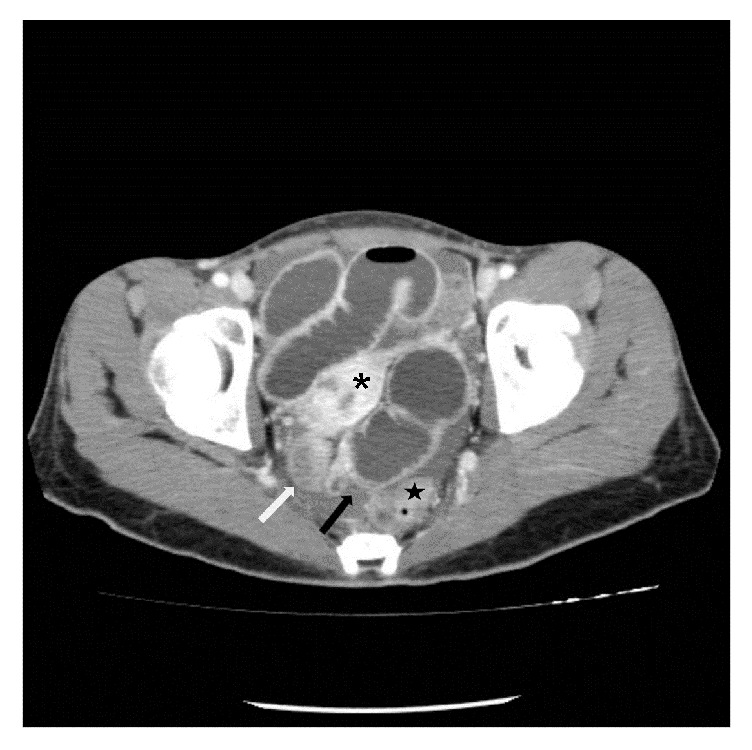
Axial CT image shows distended proximal bowel loop and collapsed distal loop (white arrow) at a transition zone (black arrow) between the uterus (asterisk) and the rectum (star).

A 26-year-old woman was admitted to the emergency department of Ilsan Paik Hospital with abdominal pain and distension lasting for 3 days. She had no history of abdominal surgery. Abdominal examination revealed mild distension and hyperactive bowel sounds. Signs of peritoneal irritation were not apparent and there were no remarkable laboratory data. Plain abdominal X-ray showed a dilated small bowel loop without free air. An emergency computed tomography (CT) revealed diffuse dilatation in proximal to mid ileal loop with abrupt luminal narrowing at distal ileum level without definite evidence of bowel ischemia (Figure 1). During admission, she was managed by intravenous hydration and placement of a nasogastric tube. However, the findings of serial simple abdomen still showed mechanical obstruction pattern without interval change, and the amount of nasogastric tube was not decreased during conservative management.

**Figure 2:**
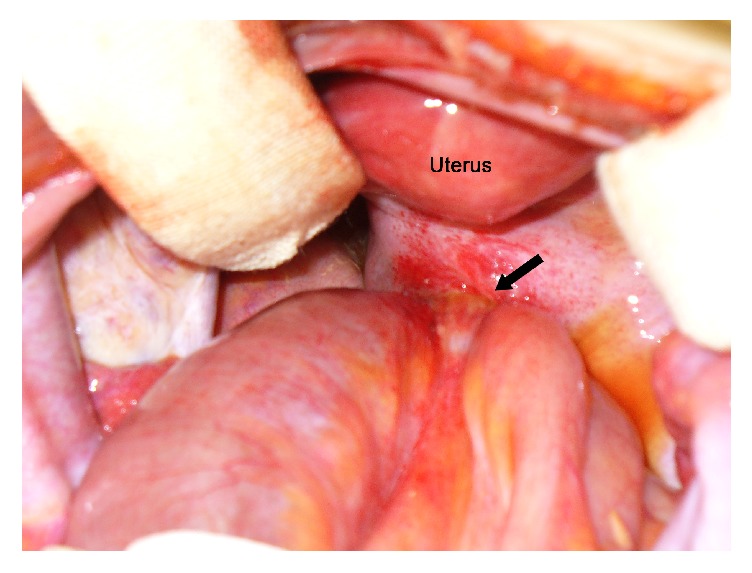
Operative findings. The distal ileum is herniated into the defect of the Pouch of Douglas (arrow).

**Figure 3:**
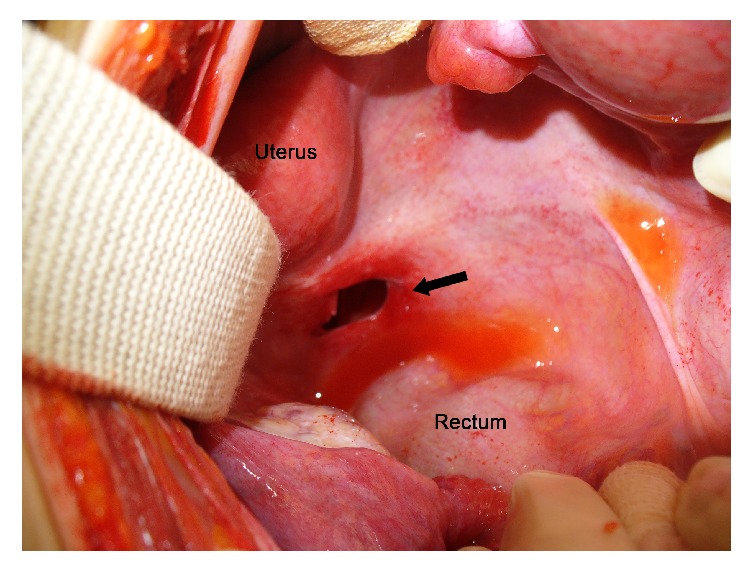
After reduction of the herniated bowel, peritoneal defect between the uterus and the rectum is shown (arrow).

Without improvement of symptoms and physical findings during conservative management, the patient underwent an exploratory laparotomy on 3rd hospital stay. During the operation, approximately 5 cm length of the distal ileum (45 cm from the ileocecal valve) had been herniated into a defect of the Pouch of Douglas, and there were no specific findings of pelvic examination (Figure 2). After reduction of the herniated bowel that was incarcerated into a defect of the Pouch of Douglas, herniorrhaphy with primary sutures of the defect site was performed (Figure 3). The postoperative course was uneventful, and the patient was discharged on the 8th postoperative day.

## 3. Discussion

In patients without history of abdominal surgery, causes of bowel obstruction such as colorectal cancer, inguinal hernia, intussusception, volvulus, and inflammatory bowel disease might be diagnosed based on physical examination and imaging studies preoperatively. However, the preoperative diagnosis of internal hernia may be difficult although preoperative diagnosis through CT has been reported [1, 8].

Congenitally created peritoneal defect and foramen may lead to internal hernias [2]. Among them, paraduodenal hernia has been reported as most frequently encountered congenital internal hernia. Transomental hernia, foramen of Winslow hernia, transmesenteric hernia, and broad ligament hernia comprise small fraction of congenital internal hernias [9]. Perineal hernia is one of the rare hernias which can occur anteriorly or posteriorly to the superficial transverse perineal muscle. Even though the precise mechanism for the development of perineal hernia is unknown, failure of regression of the peritoneal cul de sac of the embryo and the attenuation of the pelvic floor by various contributing factors have been suggested [10].

The Pouch of Douglas is an anterior peritoneal reflection between the uterus and the rectum which is so-called rectouterine pouch. Multiparity, old age, and history of pelvic surgery may lead to weakening or defect of the pelvic floor resulting in herniation of bowel though the Pouch of Douglas [10]. This type of hernia has been referred to as Pouch of Douglas hernia, and it has been described as the entity of perineal hernia [4]. However, the present case is a hernia through the peritoneal defect of Pouch of Douglas. Perineal hernia refers to the hernia caused by weak pelvic floor. But, contrary to perineal hernia in the literature, the present case is not the hernia caused by weak pelvic floor but by the defect of the Pouch of Douglas. So, the hernia in the present case may not be synonymous to perineal hernia in the literature. Small bowel hernia through the defect of the Pouch of Douglas is extremely rare whether it is congenital or acquired. To our knowledge, only 4 cases have been reported in the English literature. The cases are summarized in Table 1 [4–7]. Fiirgaard and Agertoft fist reported a 17-year-old girl presenting with incarcerated small bowel hernia into the congenital peritoneal defect of the Pouch of Douglas [5]. Peritoneal defect of the Pouch of Douglas was congenital in 2 patients and acquired in 1 patient who had a history of hysterectomy. The main symptom of perineal hernia is discomfort in sitting position and physical examination may reveal a perineal, labial, or gluteal mass. Since the hernia sac neck tends to be wide, strangulation of herniated bowel is rare [8]. However, contrary to the symptoms of typical perineal hernias, those of patient with hernia through a defect of the Pouch of Douglas were small bowel obstruction-related symptoms due to incarceration or strangulation of bowel through the peritoneal defect.

The definitive diagnosis usually has been made during the surgery, although CT findings of hernia through a peritoneal defect of the Pouch of Douglas have been reported, in which a cluster of collapsed small bowel loops in the peritoneal defect between the rectum and the uterine cervix may be detected [7]. In the present case, CT showed collapsed bowel loops between the rectum and uterus. The finding of loops between the rectum and uterus may be detected in patient with history of low anterior resection or hysterectomy, and collapsed bowel segment between the rectum and uterus accompanied by proximal small bowel dilatation may give a clue for diagnosis of hernia through a peritoneal defect of the Pouch of Douglas.

The surgical treatments of perineal hernia consist of reduction of hernia sac, ligation, excision, and approximation of the uterosacral ligament, or obliteration of hernia sac using continuous sutures through the posterior wall of the cervix and the anterior wall of the rectum [10]. However, since the mechanism of the present case and perineal hernia in the literature may be different and there was no hernia sac, closure of the peritoneal defect would be enough treatment option. Small bowel resection may be performed according to the bowel viability. Of 4 cases with hernia through a peritoneal defect of the Pouch of Douglas, small bowel resection and anastomosis were performed in 1 case. After reduction of incarcerated small bowel, primary repair of defect site was performed in 2 cases like the present case, and laparoscopic repair was performed using mesh in 1 case.

The preoperative diagnosis of internal hernia may be elusive because of the rarity of the disease entity, especially unusual type of internal hernia. Delayed diagnosis and treatment could result in morbidity and mortality. Thus, even though hernia through a peritoneal defect of the Pouch of Douglas is an extremely rare type of internal hernia and its preoperative diagnosis is difficult, a high degree of suspicion based on CT findings and history of abdominal surgery may be necessary for prompt management.

## Figures and Tables

**Table 1:** Clinical features of internal hernia through a defect of the Pouch of Douglas in English literature.

Author (year)	Age	Symptom	History of abdominal surgery	Diagnosis made by/at	Defect of the Pouch of Douglas	Management
Fiirgaard and Agertoft (1988) [5]	17	Abdominal pain, nausea, vomiting	No	Surgery	Congenital	Reduction and primary repair of a peritoneal defect
Hoeffel et al. (1992) [6]	76	Abdominal pain, vomiting	NA	Surgery	NA	Small bowel resection and anastomosis
Inoue et al. (2002) [7]	80	Abdominal distension, vomiting	Hysterectomy	Surgery	Acquired	Reduction and primary repair of a peritoneal defect
Bunni et al. (2012) [4]	77	Pain in right groin	No	Surgery	Congenital	Laparoscopic mesh herniorrhaphy
Present case (2016)	26	Abdominal pain, distension	No	Surgery	Congenital	Reduction and primary repair of a peritoneal defect

NA = no available information.

## References

[1] Blachar A., Federle M. P., Forrest Dodson S. (2001). Internal hernia: Clinical and imaging findings in 17 patients with emphasis on CT criteria. *Radiology*.

[2] Newsom B. D., Kukora J. S. (1986). Congenital and acquired internal hernias: unusual causes of small bowel obstruction. *The American Journal of Surgery*.

[3] Baessler K., Schuessler B. (2000). The depth of the pouch of Douglas in nulliparous and parous women without genital prolapse and in patients with genital prolapse. *American Journal of Obstetrics and Gynecology*.

[4] Bunni J., Teichmann D., Berstock J. R. (2012). Pouch of Douglas pelvic hernia: a rare entity managed laparoscopically. *Hernia*.

[5] Fiirgaard B., Agertoft A. (1988). Internal richter's hernia due to congenital peritoneal defect. Case report. *Acta Chirurgica Scandinavica*.

[6] Hoeffel J. C., Zimberger J., Pocard B., Hoeffel C. (1992). Demonstration by computed tomography of a case of internal small bowel herniation. *The British Journal of Radiology*.

[7] Inoue Y., Shibata T., Ishida T. (2002). CT of internal hernia through a peritoneal defect of the pouch of Douglas. *American Journal of Roentgenology*.

[8] Lubat E., Gordon R. B., Birnbaum B. A., Megibow A. J. (1990). CT diagnosis of posterior perineal hernia. *American Journal of Roentgenology*.

[9] Ghahremani G. G. (1984). Internal abdominal hernias. *Surgical Clinics of North America*.

[10] Stamatiou D., Skandalakis J. E., Skandalakis L. J., Mirilas P. (2010). Perineal hernia: surgical anatomy, embryology, and technique of repair. *The American Surgeon*.

